# Online impulse buying behavior and marketing optimization guided by entrepreneurial psychology under COVID-19

**DOI:** 10.3389/fpsyg.2022.939786

**Published:** 2022-08-16

**Authors:** Pei Wang, Sindy Chapa

**Affiliations:** School of Communication, Florida State University, Tallahassee, FL, United States

**Keywords:** online shopping, marketing effect, entrepreneurial psychology, impulse buying behavior, COVID-19

## Abstract

This work aims to promote the development of the online shopping market and improve the online marketing effect of goods. First, entrepreneurial psychology and online shopping are discussed. Then, impulse buying behavior (IBB) is analyzed, and the IBB model and hypotheses of consumers are proposed under the psychological model. Finally, consumers’ IBB during COVID-19 is assessed under the psychological models. Hedonic shopping value (HSV) is a psychological factor directly affecting consumers’ IBB during COVID-19. The results indicate that COVID-19 has a specific stimulating effect on IBB. Meanwhile, the types of goods consumers buy during COVID-19 vary widely across age groups and regions. Overall, clothing is the most purchased item by consumers. This work provides the main reference for the improvement of the online commodity marketing effect and makes a crucial contribution to the development of the online shopping market.

## Introduction

Consumers’ shopping methods are constantly changing with the popularity of network technology. Nowadays, online shopping has become the primary shopping method occupying the majority of the consumer market (Lim and Kim, 2020), especially during the Coronavirus Disease 2019 (COVID-19) epidemic. The e-commerce industry has significantly stimulated users’ impulse buying behavior (IBB) and has become essential support for the market economy (Lins and Aquino, 2020), which many studies have proved.

Luo et al. (2021) pointed out that with the rapid development of society and the economy, the purchasing behavior of consumers had changed significantly. These changes were mainly reflected in a rapid decrease in planned purchases and a rapid increase in unplanned (emotional) purchases. Consumers’ buying behavior driven by such emotional factors was called impulse buying (Luo et al., 2021). Iyer et al. (2020) used the stimulus-organism-response theory to classify the external stimuli that constitute the shopping festival atmosphere into interactivity, personalization, entertainment, and economy. They also analyzed consumers’ IBB in the festive atmosphere of online shopping by constructing a research model that affects IBB. Finally, scholars found that the personalization, entertainment, and economy of the shopping festival atmosphere have significant positive effects on consumers’ arousal emotions. In addition, interactivity, entertainment, and economy significantly positively impact consumer pleasure (Iyer et al., 2020). In addition, arousing emotion significantly affected consumers’ perception of happiness. Rachmat (2022) reported that with the development of e-commerce, the size of the online advertising market is growing by more than 50% annually. Online advertising could spark another shopping spree. Therefore, advertising needs further discussion to support online market development (Rachmat, 2022). Zafar et al. (2021a) believed that impulse buying was a hot issue in consumer behavior, with various influencing factors. Previous scholars found that many factors affected IBB, mainly focusing on personality traits and behavioral situations. Psychological conditions significantly affect human cognition, decision-making, and behavior. Psychology plays a vital role in every stage of buying. Some scholars regard psychological and situational factors as an important part. The current conclusion are immature, and further research is needed to test the hypothesis.

Firstly, the basic connotation of entrepreneurial psychology and online shopping is discussed. Secondly, the psychological factors that affect consumers’ purchasing behavior are analyzed, psychological models are established, and some hypotheses are put forward. Finally, this work comprehensively assesses a user’s IBB based on a mental model.

## Research theory and method

### Entrepreneurial psychology

**FIGURE 1:**
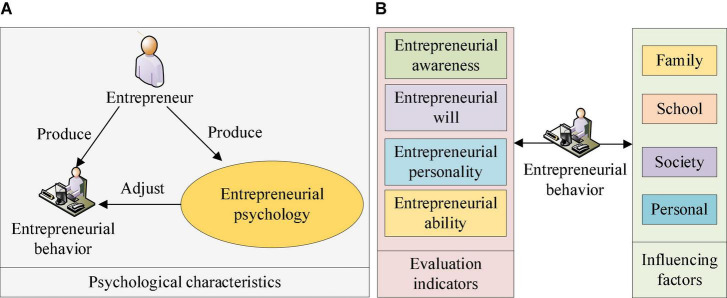
Basic situation of entrepreneurial psychology. **(A)** The basic connotation of entrepreneurial psychology; **(B)** The influencing factors and evaluation indicators of entrepreneurial psychology.

Entrepreneurial psychology refers to the psychological characteristics when entrepreneurs adjust their behavior when starting a business. On the one hand, a proliferation of people chooses to learn entrepreneurial knowledge in a university education environment due to the current socio-economic situation and the continuous development of education (Silva and Baed, 2020). On the other hand, the number of people in higher education continues to increase, worsening the problem of personal employment. Employment difficulties have become a universal social problem. As a result, many people choose to start their businesses. However, entrepreneurship is very demanding. The premise of successful entrepreneurship is entrepreneurs’ excellent qualities, including learning and psychological quality. Among them, psychological quality is the most significant (Newman et al., 2021). Many reasons affect the psychological quality of entrepreneurs, including family, society, school, and individual. These influences cannot be ignored. The evaluation indicators of entrepreneurial psychology include entrepreneurial awareness, entrepreneurial will, entrepreneurial personality, and entrepreneurial ability. Therefore, the discussion on entrepreneurial psychology is quite important (Su et al., 2020). Figure 1 shows the basics of entrepreneurial psychology.

As shown in Figure 1, entrepreneurial psychology covers many contents with a critical role. Entrepreneurial psychology refers to the personality and psychological characteristics regulating the behavior of entrepreneurs throughout entrepreneurial practice. With the further development of the market economy, many entrepreneurs are involuntarily pushed into the wave of entrepreneurship. However, entrepreneurship is not plain sailing. Whether entrepreneurship can succeed is related to entrepreneurs’ comprehensive quality and entrepreneurial psychological quality. Solid entrepreneurial psychology includes the following points:

1) Confident and decisive psychology. Self-confidence is the foundation for an entrepreneur to start a business. When choosing the path of entrepreneurship, entrepreneurs have powerful self-confidence and believe in success. At the same time, self-confidence and decisiveness are closely linked. On top of self-confidence, entrepreneurs must also be decisive.
2) Teamwork psychology. Entrepreneurs do not lack entrepreneurial heroes in their hearts and are aware that almost all entrepreneurs have experienced teamwork and hard work in the early stage of entrepreneurship.
3) Pioneering and innovative, competitive, and enterprising psychology. Entrepreneurship is starting one’s own business from scratch. In today’s fierce social competition, competitive consciousness gives entrepreneurs the possibility of social survival as a novice. Entrepreneurs must calmly consider the importance of pioneering and enterprising entrepreneurship.
4) Optimism. Entrepreneurship is a long and tortuous process. In this process, an optimistic and cheerful attitude enables entrepreneurs to maintain a happy mood. Especially in the face of setbacks and adversity, entrepreneurs may bear the great psychological pressure and produce all kinds of pessimism and disappointment. At this time, entrepreneurs must deal with all kinds of adverse situations with an optimistic and cheerful attitude and be calm. Importantly, they must believe that more joy follows the test and success.

At present, many people participate in entrepreneurship and promote the diversified development of the social economy. Entrepreneurs have become the main body of emerging economic interests in the free capital market, and entrepreneurship has become one of the main driving forces of economic development (Obschonka et al., 2020). Under the condition that entrepreneurial psychology promotes economic development and guides people to increase consumption behavior, the research on consumer psychology has become an innovative project. Entrepreneurial psychology research on consumer behavior can provide important theoretical support for optimizing online marketing. Entrepreneurial psychology is the psychological characteristic that entrepreneurs adjust their behavior throughout entrepreneurship. Under the current social situation, colleges and universities have expanded yearly enrollment. Therefore, more graduates increase the employment pressure, stimulating independent entrepreneurship under such circumstances.

### Development of online shopping

At present, various social media software and web pages have emerged and are frequently used in people’s daily life with the advance in network technology. Shopping through these channels is becoming even more common. According to a report from a consulting company, as early as 2015, the number of users of a social network-based online mall reached about 150 million, a year-on-year growth rate of 19.1%. The number of online shopping per capita was 7.2 times, a year-on-year increase of 1.2 times. The per capita online shopping amount was up to 2,134 CNY, a year-on-year growth of 75.5% (Naseri, 2021). These data indicate that online shopping has developed rapidly and has become one of the major ways of shopping for users. Moreover, consumers’ acceptance of online shopping has advanced by leaps and bounds. The emergence of online shopping is mainly due to the growth of the economy and the development of technology, which has promoted the psychology of consumers. Entrepreneurial psychology is the main reason for economic and technological development. Therefore, the research on consumer behaviors *via* entrepreneurial psychology is of great significance (Koch et al., 2020).

**FIGURE 2:**
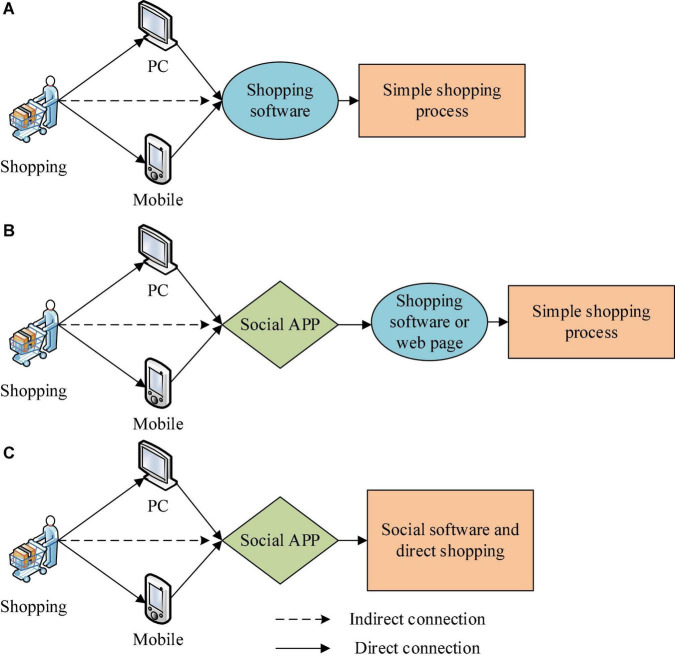
Basic forms of online shopping. **(A)** Online shopping malls; **(B)** Shopping channels *via* social software; **(C)** Shopping function of social software.

There are currently three ways to shop online. The first one is direct purchase in online shopping malls, with single content and less user-oriented publicity. The second is the shopping channel built by particular social software, which has a better marketing effect. The third is the shopping function that comes with social software. Every day, 172 million WhatsApp users send messages to WhatsApp Business accounts (sellers) to conduct transactions. It can be seen that more and more people are shopping through social software (Ali, 2020). Figure 2 shows the basic forms of online shopping.

As shown in Figure 2, online shopping focuses on improving the psychological experience of consumers to increase sales. In particular, the three shopping forms have a specific impact on network optimization, and the third method has the best optimization effect. The research on online shopping reported here can provide a necessary reference for network optimization. The third shopping method can effectively improve the marketing effect of online shopping products.

### Impulse buying behavior

Impulse buying behavior is a sudden, compulsive, and hedonistic complex buying behavior. In this kind of behavior, the rapidity of shopping decisions prevents consumers from considering comprehensive information and alternative choices. Impulse buying theory is based on consumer decision-making theory, which analyzes impulse shopping from the perspective of emotion. This view assumes that consumers may associate some highly participatory emotions, such as joy, love, fear, hope, sex, and fantasy, with shopping behavior. Because many impulsive consumers are emotion-driven, they do not conduct careful search and thoughtful evaluation before shopping but impulsively buy many unplanned goods. Even if consumers are in a negative emotional state, once they enter the store, they may be refreshed and spend more than expected. All these mean that impulse shopping is largely an unconscious buying behavior.

**FIGURE 3:**
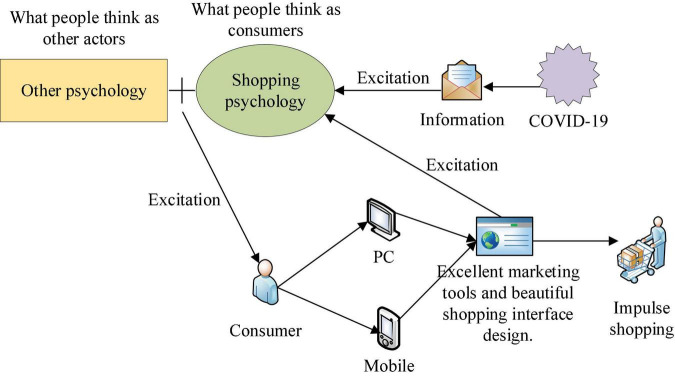
Specific design ideas of marketing plans by exploring consumers’ impulse buying behavior (IBB).

Due to economic development and great changes in shopping methods, many consumers will experience IBB. Marketers worldwide have recognized the importance of IBB. In the past 70 years, researchers have explored this phenomenon extensively. Consumption behavior can be divided into planned, unplanned, or impulsive (Rahanatha et al., 2022). Impulse consumption is closely related to hedonic shopping value (HSV) emotions, such as fun, novelty, and praise from others. In addition, the economic development level can adjust the relationship between factors (website visual attractiveness, ease of use, price, promotion, pleasure, and positive emotion) and online impulse buying (OIB) (Lee et al., 2021). In addition, people can make impulse purchases during emergencies and crises such as COVID-19. The severity of the epidemic has had a positive impact on impulse buying in China. By exploring the main consumption behaviors of consumers during COVID-19, an effective marketing plan is proposed to increase the online marketing effect of products and increase sales (Wu et al., 2020). Figure 3 shows the specific design idea of the marketing plan by exploring consumers’ IBB.

As shown in Figure 3, shopping psychology refers to the requirements to ensure the safety of the preferred products, especially food, drugs, cleaning supplies, sanitary supplies, electrical appliances, and vehicles. The psychological content of other psychological descriptions is the specific psychology to meet personal unique hobbies and tastes. The offline shopping mode was limited during COVID-19, so the online shopping mode became the first choice, which provided an excellent generation environment for the growth of shopping psychology. Moreover, COVID-19 will partly impact consumer psychology. In the context of the epidemic, consumers will reduce offline consumption activities. Constricted by living requirements, they can only consume online. Online consumption behavior can reduce contact infection, thus continuously intensifying consumers’ psychological preference for online consumption. This shopping mode can significantly stimulate users’ shopping behavior. Therefore, it is necessary to study users’ OIB to improve the marketing effect of products.

### Optimization strategy of marketing

This work optimizes the service marketing mix strategy. Among the many service marketing mix strategies, shopping websites lack an accurate grasp of the core demands of products, the design of service products, relatively complete channels, diverse promotion methods, effective management and control of service processes, a competitive physical environment, and sufficient service personnel, and customers. Correspondingly, the cost of retaining brand members increases.

The competition in the e-commerce industry is fierce. Shopping websites must adopt a refined strategy to deal with competitors. The competition of e-commerce platforms tends to be intensified: the choice of suppliers increases; the cost of potential individual merchants to open a store is reduced; the physical store is showing signs of revival; buyers face more choices on platforms and channels. Shopping platforms are ready to replace shopping brands with their dominant platforms and differentiated services. Against the fierce competition environment, shopping platforms must identify short-term and long-term competitors, create selling points in services, form the attractiveness of a tangible environment on the platform, and have strong means to retain customers. Short-term breakthrough service innovation can only reconstruct service elements and conditions, failing to innovate products, accumulate advantages, and improve the environment to face the competition of shopping websites. The same service marketing elements built by breakthrough service innovation lose significance. The comprehensive review shows that the shopping platform service marketing environment is suitable for optimizing the incremental service marketing strategy.

## Theoretical framework and hypothesis

Psychological models are adopted for the identification of the main influencing factors of IBB. The model derived from environmental psychology is adopted to simulate the context of IBB of Chinese and American consumers. Based on the psychological analysis of consumers, the psychological factors of consumers buying behaviors are classified into the following aspects.

### Hedonic shopping value

Hedonic shopping value refers to an experiential, emotional, and irrational value. It reflects the value gained from the multi-sensory, fantasy, and emotional aspects of the shopping experience. Hedonic-motivated consumers seem to derive satisfaction from shopping, leading to IBB. According to psychological models, an individual’s emotional response to the environment determines their behaviors. In other words, when consumers receive a valid response, they change their decision. Existing literature on IBB shows the direct influence of HSV on IBB. Besides, emotional responses may stimulate IBBs. The relationship is replicated in traditional shopping environments and OIB (Musadik, 2021). The following hypothesis is made based on the above.

H1: HSV has a positive effect on OIB.

### Perceived enjoyment

Perceived enjoyment (PE) refers to the level of satisfaction that customers feel when they conduct an online transaction on a website, in addition to any performance consequences that can be expected. It is vital in the process of user technology acceptance, especially for hedonic systems. Consumers with high hedonic value (HV) can get satisfaction and enjoyment from immediate happiness (Feng and Yin, 2021). Under the psychological model, PE as an organism leads to a response, namely, IBB. Hence, the following hypothesis is proposed.

H2: PE has a positive effect on OIB.

People with high HV are likely to seek enjoyment and fun through shopping. Hedonic-motivated consumers also seek acceptance and affection through shopping, including in an online shopping environment with no time and place constraints. As a result, they probably buy without a plan, such as an impulse buying. Here comes the third hypothesis.

H3: HSV markedly affects PE.

### Perceived usefulness

Perceived usefulness (PU) is the consumer’s perception of the outcome of the shopping experience. It is defined as an individual’s belief that using new technology can improve consumer performance. Extensive research has focused on the interaction between cognition and emotion, whereby cognition affects emotion and ultimately determines behavior. According to the psychological model, when consumers shop online, the usefulness they perceive from the Internet can lead to some entertainment organisms (Wang, 2021). Here comes the fourth hypothesis.

H4: PU has a positive effect on OIB.

### Website stimulation: Ease of navigation and website appearance

Website stimulation is one of the external factors of IBB, representing the marketing cues or incentives set controlled by marketers to attract the IBB of consumers. The shopping site environment stimulating OIB includes the website appearance (WA), ease of navigation (EN), payment security, and exclusive online discounts. EN means the smooth interaction between the consumers with the shopping website. Navigation is the most common interaction channel between consumers and web applications. WA includes the colors, fonts, images, and overall style of the shopping website. These two variables have positive effects on PU, HV, and IBB. Based on psychological models, when consumers easily browse a website and find its appearance attractive, they are likely to view the product as applicable and have a high HV (Conversano and Giuseppe, 2021). The following two hypotheses are proposed.

H5: EN has positive effects on HSV, PE, and PU.

H6: WA has positive effects on HSV, PE, and PU.

### Related timely reminder: The pandemic COVID-19

Most existing studies focus on preventative health behaviors rather than consumer behaviors. Nevertheless, the unique and unexpected economic situation brought about by the COVID-19 pandemic calls for a close examination of how several external cues affect impulse buying. In the context of communication, a cue is a signal to the audience or consumer due to the information received during quarantine preparations. The information related to pandemic cues (PC) such as time constraints, product scarcity, shortages, and other stimuli are sent to consumers (Chopdar et al., 2022). Epidemic cues can affect online consumers psychologically, which is predicted here. This work also proposes an undirected hypothesis.

H7: PC can signally affect OIB.

**FIGURE 4:**
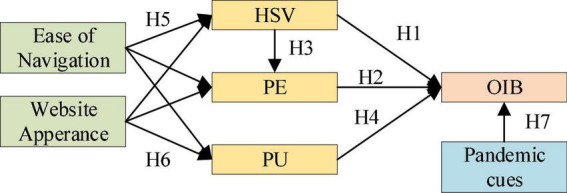
Psychological models of impulse buying behavior (IBB).

Figure 4 reveals the relationships mentioned by all the above assumptions.

All the hypotheses are proposed based on IBB and are mutually related (as shown in Figure 4), indicating the preciseness of this work.

### Product category

While many studies mention the most popular products among consumers during the COVID-19 pandemic, empirical research on impulsive-buying products is lacking. Additionally, from the previous studies, demographic factors are predictors of the consumer’s impulsiveness. Consumer characteristics and demographic factors can affect IBBs, which are also affected by local market conditions and various cultural forces. A cross-cultural study predicts that the age of Israeli and American consumers is inversely related to impulsivity characteristics. It explores product categories during the pandemic and tests region or age as a demographic factor for product impulse consumption (Zafar et al., 2021b). Based on this, the following questions are proposed.

RQ1: which products are more likely to be purchased online during the COVID-19 outbreak in China?

RQ2: is it possible to observe age variables across product categories through OIB during the COVID-19 outbreak in China?

RQ3: does geographic location have an impact on OIB?

## Research design and method

This is a multi-stage, multi-method study. Firstly, a focus group is formed to identify projects for “epidemic cues” measurements and product categories. Ten college students participated in the project identification. The statements of item selection were recorded, and three items of the “epidemic cue” factor were obtained (Cruz Villazón et al., 2020).

Subsequently, an online survey was performed by using a convenient sample from China to test the conceptual model. The survey sent invitations to Chinese participants and asked them to send the survey invitation to someone they knew. The questionnaire includes five questions per page. At the top of each page is a statement: “When answering the following question, please try to recall your last online shopping experience during the COVID-19 pandemic.” The following statement is used to measure buying behavior: “When answering the following question, please recall the last item you purchased online during the COVID-19 pandemic.” Verification questions were also included in the survey.

Data are collected from September 15 to 21, 2020. As of September 22, the number of positive cases of Corona Virus Disease 2019 (COVID-19) has risen steadily over the past 30 days, with fewer than 30 cases per day. During the data collection period, the development of COVID-19 in China was generally under control. Three hundred twenty-two samples are collected. After excluding outliers, missing data, and unreliable cases (*n* = 90), 232 samples are included in the survey. Females (125) accounted for 53.9%, and males (107) accounted for 46.1%. Most of the participants were from central China with 79.3%, followed by western China (12.5%) and eastern China (8.2%); 26.7% (*n* = 62) of the participants were in the 25–30 age group, the largest age group in this survey, followed by the 31–40 age group (23.7%) and the 51–60 age group (22.41%). Due to the geographical limitation of data collection, most of the research subjects were from the central region of China. The relevant test data may appear to be biased due to regional characteristics. This work employs seven developed and validated scales to measure variables in the model to reduce experimental bias. Additionally, outbreak clues created for this work are measured. All measurements are performed using a seven-point Likert scale (Santini et al., 2019; Shahpasandi et al., 2020). In addition, population issues are also included. These items are shown in Table 1.

**TABLE 1:** Test criteria and the coefficients.

Item	Item load	Cronbach’s α coefficient
**Factor 1: EN (Taylor et al., 2010)**		
Navigating these web pages where I purchased the item mentioned above was easy.	0.82	0.93
My interaction with the website where I purchased the item was clear and understandable.	0.88	
It is easy to become skillful at navigating the pages of the website where I purchased the item.	0.86	
Overall, the pages where I purchased the item were easy to navigate.	0.89	
It was pleasant to follow the overall flow of the website where I purchased the item.	0.86	
It was pleasant to follow and use the menu structure of the site where I purchased the item.	0.85	
**Factor 2: WA (Taylor et al., 2010)**		
The shopping site where I purchased the item was visually pleasing.	0.82	0.95
The shopping site where I purchased the item displayed a visually pleasing design.	0.87	
The shopping site where I purchased the item was visually appealing.	0.92	
The images and typographies used in the shopping sites where I purchased the item were stylish.	0.92	
The overall atmosphere and screen display of the shopping sites where I purchased the item were well coordinated.	0.91	
It was pleasant to see the provided information on each screen of the shopping site where I purchased the item.	0.86	
**Factor 3: HSV (Babin et al., 1994; Griffin et al., 2000)**		
This online shopping experience was truly a joy during this hard time.		
Compared to other things I could have done, the time spent online shopping was truly enjoyable.	0.72	0.88
I enjoyed online shopping for its own sake, not just for the items I may have purchased.	0.84	
During my online shopping, I felt the excitement of the hunt.	0.87	
While I was online shopping, I felt a sense of adventure.	0.88	
**Factor 4: PE (Chang and Cheung, 2001)**	0.75	0.92
My interaction with the product(s) purchased online during the COVID-19 pandemic was enjoyable.	0.93	
My interaction with the product(s) purchased online during the COVID-19 pandemic is exciting.	0.92	
My interaction with the product(s) purchased online during the COVID-19 pandemic is pleasant.	0.93	
**Factor 6: PU (Davis, 1989)**		
Using these product(s) I purchased online, I can improve my performance in life during the COVID-19 pandemic.	0.87	0.86
Using these product(s) I purchased online, I can increase my productivity during the COVID-19 pandemic.	0.84	
Using these product(s) I purchased online, I can enhance my effectiveness in daily life during the COVID-19 pandemic.	0.91	
I would find the product(s) I purchased online useful in my life during the COVID-19 pandemic.	0.76	
**Factor 7: PC (self-developed)**		
I bought the product(s) listed above because of the COVID-19 pandemic.	0.87	0.87
I bought the product(s) listed above primarily because of the COVID-19 pandemic.	0.94	
I would not have bought the product(s) listed above if it was not for the COVID-19 pandemic.	0.87	
**Factor 8: OIB (Rook and Fisher, 1995)**		
During the COVID-19 pandemic, I had the urge to purchase items other than or in addition to my specific shopping goal.	0.91	0.90
During the COVID-19 pandemic, I had a desire to buy items that did not pertain to my specific shopping goal.	0.93	
During the COVID-19 pandemic, I had the inclination to purchase items outside my specific shopping goal.	0.91	

N = 232.

## Psychological assessment of impulse buying behavior

### Test for the pandemic cues scale

Exploratory factor analysis was performed to validate the PC scale measurements to identify one-dimensional measures, test convergence, and distinguish validity. After removing the one-dimensional measurement items, the load of a single item is 0.7 on each scale, with one on the WA scale and two on the HSV scale. Subsequently, Cronbach’s α coefficient was used to estimate the cross-structure to test questionnaire reliability, and the value should be higher than the recommended 0.7. Table 2 summarizes all the items that meet this standard. Eventually, the mean variance of each variable reaches 0.50. The numerical differences between each construct and its indicator variance-ratio and error variance vary widely. Table 2 shows the results of the PC scale.

**TABLE 2:** Correlation and average variance of pandemic cues (PC).

	Mean-variance	PE	PC	EV	WA	HSV	PU	OIB
PE	0.797	**0.893**						
PC	0.709	0.148	**0.842**					
EV	0.691	0.625	0.148	**0.831**				
WA	0.751	0.711	0.268	0.837	**0.867**			
HSV	0.611	0.716	0.310	0.434	0.543	**0.782**		
PU	0.631	0.710	0.435	0.638	0.679	0.611	**0.794**	
OIB	0.758	0.506	0.292	0.155	0.365	0.712	0.367	**0.870**

The bold value represents the peak value for this test.

### Verification of hypotheses

Structural equation modeling (SEM) was run using Arbuckle-Marcoulides-Schumacker to test conceptual models and hypotheses. All variables except the deleted items were included in the SEM analysis. According to the results, these values showed a good fit. For instance, χ^2^/*df* ≤ 3.0, the comparing fit indicator (CFI) ≥ 0.90, the goodness of fit indicator (GFI) ≥ 0.90, the Tucker-Lewis indicator (TLI) ≥ 0.90, and root mean square error average (RMSEA) ≤ 0.80. However, the fitting indicator of the theoretical model did not reach the recommended level: χ^2^/*df* = 3.096, CFI = 0.864, GFI = 0.762, TLI = 0.850, and RMSEA = 0.095. After the rejected relationship was deleted, the alternative model was investigated through the modified indicators. Each indicator for the nested model was improved to meet the standards: χ^2^/*df* = 2.899, CFI = 0.887, GFI = 0.772, TLI = 0.875, and RMSEA = 0.091.

**FIGURE 5:**
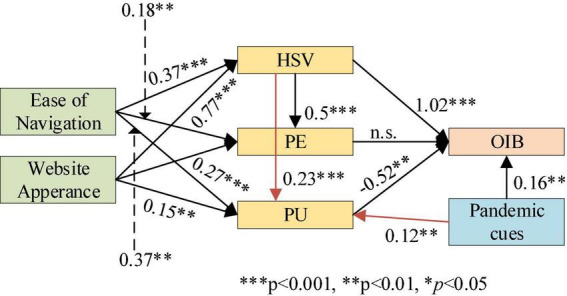
Hypothesis verification results of the psychological model.

Two new relationships were proposed for the alternative model: the relationship between PC and PU and that between HSV and PU. The results suggest that nine hypotheses are established (H1, H3, H5a, H5b, H5c, H6a, H6b, H6c, H7), and the two relations are not established, that is, PE of OIB (H2) and positive PU of OIB (H4). Specifically, H2 does not hold because of insufficient coefficient paths, while H4 does not because regressions are intrinsically correlated. Additionally, PU has an obviously negative effect on OIB (*P* < 0.01) (Figure 5).

Figure 5 presents the verification results of the relationship among the hypotheses of the psychological model. During the qualitative phase, 13 categories were identified as purchased during the pandemic [books, electronics, cosmetics, and personal care products, video games and consoles, household tools and hardware, pet-related items, compact disk (CD), and digital video disk (DVD), mobile phone and tablet accessories, household appliances, kitchen tools, home gardens, clothing, and sports equipment]. A correlation analysis was performed to investigate the relationship between product categories and PC. Clothing was remarkably associated with PC (*r* = 0.17, *P* < 0.001).

The participants’ overall product consumption was estimated to explore whether PC affected consumers’ cross-category consumption. The cluster analysis was conducted to identify the consumption categories. The created categories include individually consumed products (books, clothing, personal care, and sports equipment), products consumed by groups/households (household tools and hardware, pet-related items, home gardens, etc.), and electronic products for individual use or for families/groups (electronics, video games, and game consoles, etc.). Changes in 5 product categories related to products purchased online by participants were observed. The statements of consumers are as follows. (1) All products they purchased are for personal use only. (2) Electronic products they purchased are for personal use only. (3) All products they purchased are for family and group use. (4) Purchased products are for all to use. (5) All three categories (personal, home/Group, electronic) of the products are purchased.

An Analysis of Covariance was carried out to explore the effect of age-specific IBB on product categories. The results indicate a considerable difference among the five groups (*P* < 0.001). Besides, all age groups (under 25, 25–30, 31–40, 41–50, 51–60) are the most likely to impulse buy household and group items, followed by personal items and for personal use or home/groups used, and finally electronics. Tables 3, 4 show the specific results.

**TABLE 3:** Age effects.

	Sum of squares	*df*	Mean square	*F*	Sig.
Between-group	60.03	5	12.01	7.66	0.000
Within-group	354.25	226	1.567		
Total	414.27	231			

**TABLE 4:** Product category’s effects.

	Personal use only (1)	Electronic product (2)	Family use products (3)	Personal and family use products (4)	Three categories (5)
*M*	2.93_*b*_	2.63_*b*_	4_*a*_	2.85_*b*_	2.84_*b*_
*SD*	1.26	1.31	1.15	1.09	1.21

F(5,226) = 7.66, *P* < 0.001; a and b represent the two types in H6. The data with no subscript were in common differences, *P* < 0.05; Bonferroni for post-hoc comparisons: b.

Furthermore, a One-Way Analysis of Variance was performed for the geographic location of participants in the five consumption categories by using a single factor. The results indicate a considerable difference among the five groups (*P* < 0.05). In other words, impulse buying during the COVID-19 pandemic varies across geographic regions. Participants in various regions (such as eastern China, western China, central China, and northeastern China) reported that they had bought all three categories. Tables 5, 6 show the effects of geographic location and the product categories.

**TABLE 5:** Effects of geographic location.

	Sum of squares	*df*	Mean square	*F*	Sig.
Between-group	2.84	5	0.57	2.72	0.021
Within-group	47.43	226	0.21		
Total	50.27	231			

**TABLE 6:** Effects of product categories.

	Personal use only (1)	Electronic product (2)	Family use products (3)	Personal and family use products (4)	Three categories (5)
*M*	2.01_*b*_	2.00_*b*_	2.08_*a*_	2.15_*b*_	2.29_*b*_
*SD*	0.40	0.51	0.36	0.37	0.69

F(5,226) = 2.72, *P* < 0.05; a and b represents two types in H6. The data with no subscript were in common differences, *P* < 0.05; Bonferroni for post-hoc comparisons: b.

To sum up, nine conceptual models of 11 hypotheses (H1, H3, H5a, H5b, H5c, H6a, H6b, H6c, H7) to predict the OIB behaviors of Chinese consumers hold. Nonetheless, the association between PE and OIB (H2) was rejected. The model reported here shows a negative correlation between PU and OIB, leading to the rejection of H4. Moreover, the alternative model proposes two new relationships: PC has a positive effect on PU, and HSV has a positive effect on PU.

Out of 13 product categories, clothing was the most purchased product in 13 product categories by Chinese consumers during the COVID-19 outbreak. Household products were the most likely to be impulse buys among different age groups in the Chinese sample. The top three categories of home furnishing consumers were geographically dispersed.

### Discussion

This work proposes IBB and marketing optimization strategies based on the development of entrepreneurial psychology and online shopping. Supported by the shopping framework theory, this work uses shopping psychology to develop the psychological measurement scale of online shopping during COVID-19. In contrast, Cawley et al. (2019) studied the impact of cross-border shopping and consumption. The results showed that the tax increase in retail prices varied from jurisdiction to jurisdiction, ranging from 50 to 100% lower than the tax. Larson and Shin (2018) studied the influencing factors of shopping convenience and shopping behavior to link fear during natural disasters, shopping convenience, and shopping behavior. The results would help retailers and service providers understand shopping motivation and develop strategies to deal with natural disasters. Müller et al. (2021) examined object attachment psychology in shopping disorders. They found that shopping disorder psychology was mainly driven by the recognition of material value and the regulation of negative emotions through purchasing material goods. Hasan et al. (2019) investigated the entrepreneurial ability of college students. The results corroborated that entrepreneurial learning significantly impacted the entrepreneurial students’ positive psychological capital. Positive psychological capital significantly impacted students’ entrepreneurial ability. Su et al. (2020) surveyed entrepreneurial motivation and entrepreneurial process from the perspective of positive psychology. They tried to interpret the continuous incentive mechanism of entrepreneurs from the perspective of the dynamic process of entrepreneurship and positive psychology. To sum up, entrepreneurs can persist in a highly uncertain environment and improve the completion rate of entrepreneurial behavior by gaining positive emotions throughout the entrepreneurial process.

COVID-19 has made it necessary to optimize marketing strategies and bring more consumer benefits. Marketing strategy optimization should timely insight into the users’ needs in special periods and provide advanced enterprise values. Firstly, in special periods, the best marketing is to give up their short-term benefits, meet the needs of different groups through various forms of public welfare, and provide warmth and value for them. Secondly, enterprises should shift their attention from online businesses to solving users’ problems by taking advantage of the COVID-19 period and combining their professional abilities. In doing so, enterprises can expand communication, enhance brand influence, and provide practical value to customers. Finally, instead of focusing on offline consumption, enterprises or merchants can operate private domain traffic online for sales promotion to maintain their popularity.

## Conclusion

This work aims to explore the IBB of consumers during the COVID-19 outbreak and design ideal product marketing plans. The association between website stimulation associated with the OIB behavior and consumer attitudinal responses was explained based on psychological models, and the regulatory role of organisms was proposed, such as PU, PE, and HSV. With the psychological models, impulsive behavior is predicted by assuming that website antecedents are related to consumers’ HSV, PU, and PE. Most of the hypotheses were validated (H1, H3, H5a, H5b, H5c, H6a, H6b, H6c, H7). Correlations between PC and PU, as well as that between HSV and PU, were also found. Furthermore, this work attempts to fill three gaps in the current research. First, there is no comprehensive model to examine the effects of websites and epidemic stimuli on OIB behavior. Second, there are limited studies in countries other than the United States. Third, it is necessary to expand the survey of consumer impulse buying attitudes based on different stimuli. There are certain deficiencies in the research. Although comprehensive hypotheses were proposed and verified, the marketing plan design of the product is not complete enough. The future study will strengthen the marketing plan to dramatically improve the marketing effect of online shopping.

## Data availability statement

The raw data supporting the conclusions of this article will be made available by the authors, without undue reservation.

## Ethics statement

The studies involving human participants were reviewed and approved by Florida State University Ethics Committee. The patients/participants provided their written informed consent to participate in this study. Written informed consent was obtained from the individual(s) for the publication of any potentially identifiable images or data included in this article.

## Author contributions

Both authors listed have made a substantial, direct, and intellectual contribution to the work, and approved it for publication.
